# Association between serum prostacyclin and cerebrovascular reactivity in healthy young and older adults

**DOI:** 10.1113/EP090903

**Published:** 2023-05-12

**Authors:** Adam T. Corkery, Kathleen B. Miller, Carissa A. Loeper, Laura H. Tetri, Andrew G. Pearson, Nicole A. Loggie, Anna J. Howery, Marlowe W. Eldridge, Jill N. Barnes

**Affiliations:** ^1^ Bruno Balke Biodynamics Laboratory, Department of Kinesiology University of Wisconsin Madison Madison WI USA; ^2^ Department of Pediatrics University of Wisconsin School of Medicine and Public Health Madison WI USA

**Keywords:** ageing, cerebral artery blood velocity, cyclooxygenase, indomethacin, platelet activation

## Abstract

Platelet activation may contribute to age‐related cerebrovascular dysfunction by interacting with the endothelial cells that regulate the response to vasodilatory stimuli. This study evaluated the relationship between a platelet inhibitor, prostacyclin, and cerebrovascular reactivity (CVR) in healthy young (*n* = 35; 25 ± 4 years; 17 women, 18 men) and older (*n* = 12; 62 ± 2 years; 8 women, 4 men) adults, who were not daily aspirin users, before and after cyclooxygenase inhibition. Prostacyclin was determined by levels of 6‐keto‐prostaglandin F1α (6‐keto PGF1α) in the blood. CVR was assessed by measuring the middle cerebral artery blood velocity response to hypercapnia using transcranial Doppler ultrasound before (CON) and 90 min after cyclooxygenase inhibition with indomethacin (INDO). In young adults, there were no associations between prostacyclin and middle cerebral artery CVR during CON (*r* = −0.14, *P* = 0.415) or INDO (*r* = 0.27, *P* = 0.118). In older adults, associations between prostacyclin and middle cerebral artery CVR during CON (*r* = 0.53, *P* = 0.075) or INDO (*r* = –0.45, *P* = 0.136) did not reach the threshold for significance. We also evaluated the relationship between prostacyclin and the change in CVR between conditions (ΔCVR). We found no association between ΔCVR and prostacyclin in young adults (*r* = 0.27, *P* = 0.110); however, in older adults, those with higher baseline prostacyclin levels demonstrated significantly greater ΔCVR (*r* = –0.74, *P* = 0.005). In conclusion, older adults with higher serum prostacyclin, a platelet inhibitor, may rely more on cyclooxygenase products for cerebrovascular reactivity to hypercapnia.

## Abstract (highlights)

- **What is the central question of this study?**
  What is the relationship between prostacyclin and cerebrovascular reactivity to hypercapnia before and after administration of a cyclooxygenase inhibitor, indomethacin, in healthy young and older adults?
- **What is the main finding and importance?**
  Serum prostacyclin was not related to cerebrovascular reactivity to hypercapnia before or after administration of indomethacin. However, in older adults, serum prostacyclin was related to the magnitude of change in cerebrovascular reactivity from before to after indomethacin administration. This suggests that older adults with higher serum prostacyclin may rely more on cyclooxygenase products to mediate cerebrovascular reactivity.

## 1 INTRODUCTION

Cerebral blood flow increases when presented with a vasodilatory stimulus, such as an increase in CO_2_ or neuronal activation (MacKay et al., 2016). However, ageing or disease may impair the ability of the cerebral vasculature to regulate flow, and reduced cerebral blood flow responses to stimuli are associated with both cardiovascular and cerebrovascular disease (Davis et al., 1983; Galvin et al., 2010; Mandell et al., 2008; Matthews et al., 1993). Decline in cerebrovascular function, which can be assessed by quantifying the cerebral blood flow or cerebral artery blood velocity response to hypercapnia (i.e., cerebrovascular reactivity), may also precede the onset of cognitive impairment (Glodzik et al., 2013; Iadecola, 2004; Kisler et al., 2017; Richiardi et al., 2015; Silvestrini et al., 2006). Therefore, understanding the mechanisms of cerebral blood flow regulation with primary ageing may be vital to understanding the progression of cognitive decline.

There are many factors that may influence cerebrovascular function, including the bioavailability of vasoactive molecules and the structural integrity of the cerebral vessels (Salvemini et al., 2013). One such factor is elevated platelet activation. Elevated platelet activation initiates vasoconstriction and is implicated in the progression of cardiovascular disease and microvascular impairments in the brain (Raz et al., 2013; Ross, 1995). Further, we have shown that higher platelet activation was associated with reduced cerebrovascular reactivity in postmenopausal women with a history of preeclampsia (Barnes et al., 2018). Prostacyclin, a product of cyclooxygenase, is an inhibitor of platelet activation and protects against cardiovascular disease (Arehart et al., 2007). Prostacyclin is also a vasodilator, and inhibition of its synthesis (via cyclooxygenase inhibition) results in a decline in cerebral blood flow and cerebrovascular responses to vasoactive stimuli (Barnes et al., 2012).

Despite the finding of impaired cerebral artery blood velocity and cerebrovascular reactivity with cyclooxygenase inhibition, the association between circulating prostacyclin and cerebrovascular reactivity has not been evaluated in healthy adults. Further, the influence of basal levels of serum prostacyclin on cerebral blood flow regulation and cerebrovascular reactivity in the presence of a cyclooxygenase inhibitor, such as indomethacin, is unknown. Therefore, the purpose of this study was to determine the relationship between serum prostacyclin levels and cerebrovascular reactivity in healthy young and older adults before and after cyclooxygenase inhibition. We hypothesized that serum prostacyclin would be positively associated with cerebrovascular reactivity. Our secondary hypothesis was that the relationship between serum prostacyclin and cerebrovascular reactivity would be age dependent.

## 2 METHODS

### 2.1 Ethical approval

The study protocol was approved by the institutional review board at the University of Wisconsin–Madison (2015‐0331) and the study conformed to the standards set by the *Declaration of Helsinki*. All participants provided informed written consent before all study procedures.

### 2.2 Participants

Thirty‐five young (25 ± 4 years; 17 females, 18 males) and 12 older (62 ± 2 years; 8 females, 4 males) adults participated in the study. Participants were recruited using flyers and word of mouth in Dane County, WI, USA, and the surrounding area. Participants had a body mass index (BMI) <30 kg/m^2^, were non‐smoking, normotensive and excluded if they presented with history or evidence of hepatic, renal or haematological disease, peripheral vascular disease, stroke or neurovascular disease, cardiovascular disease, diabetes, or other chronic pathologies, as determined by a health history questionnaire. Additionally, participants were excluded if they took daily aspirin. Physical activity levels were assessed using the Godin–Shepherd Leisure‐Time Physical Activity Questionnaire (Godin & Shephard, 1985). Premenopausal women were studied during day 2−6 of their menstrual cycle or during the non‐active pill phase of oral contraception (*n* = 7). Young women were not pregnant as determined by a urine pregnancy test. Older women were postmenopausal for >1 year and not taking any menopausal hormone therapy. All study procedures were approved by the Institutional Review Board of the University of Wisconsin‐Madison and were performed according to the *Declaration of Helsinki*, including obtaining written informed consent from each participant prior to the study. After written informed consent was obtained from each participant, an initial screening visit was performed where participants were familiarized with all study procedures.

### 2.3 Experimental protocol

Participants were instructed to refrain from caffeine, chocolate, over‐the‐counter medications, alcohol and exercise for 24 h prior to the study visit. Additionally, participants completed an overnight fast, were not habitual aspirin users and refrained from taking any non‐steroidal anti‐inflammatory drugs (NSAIDs) for 5 days prior to data collection. Experimental procedures were conducted in a controlled ambient temperature between 22°C and 24°C. Upon arrival at the laboratory, height and weight were measured using a standard scale and stadiometer. After at least 10 min of supine rest, brachial blood pressure was measured in the supine position in triplicate and the three measurements were averaged and used to calibrate the Finapres device (see below). This protocol was followed for the blood draw and cerebrovascular reactivity testing.

### 2.4 Blood draw

On a separate day from the cerebrovascular reactivity testing, a trained phlebotomist collected 4 ml of blood from the antecubital vein by venipuncture. Samples were collected in 4 ml serum clot activator vacutainers. Sample tubes were kept at room temperature for >1 h before centrifugation. The serum was then aliquoted and stored at −80°C until analysis. Blood draws were collected on the screening day, which ranged from 1 to 114 days apart from the cerebrovascular reactivity testing.

### 2.5 Cerebrovascular reactivity testing

Participants were instrumented with a three‐lead electrocardiogram to measure heart rate and a nasal cannula to measure end‐tidal CO_2_ (ETCO_2_; Datex Ohmeda, GE Healthcare, Fairfield, CT, USA). Participants were also instrumented with a finger blood pressure cuff with a height‐correction unit to measure beat‐by‐beat blood pressure (Finapres Medical Systems, Amsterdam, Netherlands). Cerebrovascular reactivity was measured as the middle cerebral artery (MCA) blood velocity response to stepped hypercapnia. MCA blood velocity was measured using a transcranial Doppler ultrasound 2 MHz probe placed on the transtemporal window in order to insonate the MCA (Spencer Technologies (Redmond, WA, USA) ST3 Transcranial Ultrasound System). A headband was used to secure the probe so the insonation angle and position were maintained throughout the entire study protocol. For the administration of hypercapnia, participants were supine and instrumented with a mask with a one‐way valve to prevent rebreathing (Hans Rudolph Inc., Shawnee, KS, USA). Stepwise increases of 2%, 4% and 6% CO_2_ with 21% oxygen and balanced nitrogen were administered for 3 min at each level as previously described (Barnes et al., 2018; Miller et al., 2018). Heart rate, beat‐by‐beat mean arterial pressure (MAP), MCA blood velocity (MCAv) and breath‐by‐breath ETCO_2_ were recorded continuously throughout the cerebrovascular reactivity protocol. Cerebrovascular reactivity was assessed before and 90 min after cyclooxygenase inhibition with indomethacin (INDO). After control cerebrovascular reactivity measurements (CON), the cyclooxygenase inhibitor INDO was orally administered at 1.2 mg/kg with 10 ml of Maalox to reduce stomach irritation. After a 90‐min wash‐in period, where participants rested quietly, the cerebrovascular reactivity protocol was repeated. The 90 min wash‐in period was chosen based on published literature (Xie et al., 2006) and our previous work suggesting 90 min was a sufficient wash‐in period for taking cerebrovascular reactivity measures after the drug administration (Barnes et al., 2012; Taylor et al., 2014).

### 2.6 Blood analysis

Prostacyclin was determined by levels of 6‐keto‐prostaglandin F1α (6‐keto‐PGF1α) in the serum, which is a stable hydrolysis product of prostacyclin. 6‐keto‐PGF1α was measured using cell enzyme linked immunosorbent assays (ELISA; 6‐keto‐PGF1α ELISA Kit ab133023, Abcam, Cambridge, UK). Samples were run in duplicate and the coefficient of variation for the sample duplicates was 7.9%.

### 2.7 Data analysis and statistics

Data were sampled at 250 Hz and analysed offline (WinDaq, DATAQ Instruments, Akron, OH, USA). MCAv, MAP, and ETCO_2_ were averaged over the last minute of each CO_2_ level during the stepped hypercapnia protocol. Cerebrovascular reactivity was calculated, using linear regression, as the relationship between MCAv and ETCO_2_, with the last minute averages of each CO_2_ level serving as a data point. The cerebrovascular conductance index (CVCi) was calculated as MCAv/MAP and used to determine cerebrovascular reactivity as the linear relationship between CVCi and ETCO_2_ with the last minute averages of each CO_2_ level serving as a data point. Delta cerebrovascular reactivity was measured as INDO MCAv or CVCi reactivity minus CON MCAv or CVCi reactivity. Statistical analysis was performed in Sigmaplot for Windows version 13.0 (Systat Software, San Jose, CA, USA). Prior to each analysis, normality was assessed using a Shapiro–Wilk test, and equal variance was assessed using a Brown–Forsythe test. Non‐parametric statistical tests were used as appropriate. Demographic variables were compared between age groups using the Student's *t*‐test. The responses to hypercapnia (MCAv, MAP, CVCi, ETCO_2_ and cerebrovascular reactivity) were assessed using a two‐way repeated measures ANOVA (age [young vs. older adults], condition [CON vs. INDO], and age × condition interaction) followed by the Holm–Sidak method to test pairwise comparisons. Delta cerebrovascular reactivity was compared between age groups using a Kruskal–Wallis ANOVA on ranks. A Spearman rank correlation was used to assess the association between prostacyclin and cerebrovascular reactivity in all participants, in young adults only and in older adults only. Statistical significance was set a priori at *P* < 0.05.

## 3 RESULTS

### 3.1 Participants

There were no differences between age groups in height, weight and BMI (Table 1). In addition, there were no differences between age groups in brachial cuff blood pressure, heart rate at rest, or serum prostacyclin levels (Table 1).

**TABLE 1:** Participant characteristics.

Variable	Young adults (*n* = 35)	Older adults (*n* = 12)	*P*
Male:female (*n*)	17:18	4:8	
Age (years)	25 ± 4	62 ± 2	<0.0001
Height (cm)	173 ± 8	171 ± 7	0.424
Weight (kg)	70 ± 10	67 ± 13	0.403
Body mass index (kg/m^2^)	23 ± 2	23 ± 3	0.705
Systolic blood pressure (mmHg)	119 ± 9	118 ± 8	0.698
Diastolic blood pressure (mmHg)	70 ± 6	74 ± 7	0.072
Mean arterial pressure (mmHg)	86 ± 7	88 ± 7	0.313
Heart rate at rest (bpm)	55 ± 9	55 ± 7	0.706
GODIN score	53 ± 25	58 ± 26	0.602
6‐keto‐PGF1α (pg/ml)	15.5 ± 3.7	16.3 ± 3.1	0.515

Participant demographic data are means ± SD. Serum prostacyclin concentrations were assessed by measuring 6‐keto‐PGF1α. Young and older adults were compared using Student's *t*‐test.

### 3.2 Cardiovascular and cerebral haemodynamics during stepped hypercapnia

At baseline and during hypercapnia, there were no age, condition, or age × condition interaction effects on ETCO_2_ or MAP with an exception of a significant condition effect for ETCO_2_ at 6% CO_2_ (Table 2). For cerebral haemodynamics, there were no age group differences in MCAv or CVCi between young and older adults at baseline, or during any stage of hypercapnia (Table 2). After INDO, MCAv and CVCi were lower at baseline and at each level of hypercapnia compared with CON (Table 2). There were no age × condition interaction effects on MCAv or CVCi at baseline or during hypercapnia (Table 2).

**TABLE 2:** Cardiovascular and cerebral haemodynamics during stepped hypercapnia.

	Young adults		Older adults		*P*		
Variable	CON (*n* = 35)	INDO (*n* = 35)	CON (*n* = 12)	INDO (*n* = 12)	Age	Condition	Interaction
ETCO_2_ (mmHg)							
Baseline	40 ± 6	41 ± 6	41 ± 4	41 ± 4	0.844	0.821	0.492
2% CO_2_	42 ± 5	43 ± 4	42 ± 4	42 ± 3	0.866	0.460	0.249
4% CO_2_	46 ± 4	46 ± 4	46 ± 3	46 ± 3	0.888	0.880	0.418
6% CO_2_	50 ± 3	50 ± 3	50 ± 3	49 ± 4	0.325	0.016	0.371
Mean arterial pressure (mmHg)							
Baseline	91 ± 14	94 ± 13	94 ± 16	91 ± 10	0.944	0.868	0.138
2% CO_2_	90 ± 13	93 ± 13	95 ± 16	91 ± 10	0.756	0.688	0.093
4% CO_2_	92 ± 13	95 ± 13	97 ± 17	92 ± 12	0.799	0.698	0.093
6% CO_2_	94 ± 15	96 ± 14	102 ± 18	97 ± 11	0.372	0.712	0.154
MCAv (cm/s)							
Baseline	61 ± 17	42 ± 8	53 ± 19	38 ± 11	0.174	<0.0001	0.341
2% CO_2_	63 ± 18	43 ± 8	56 ± 20	38 ± 11	0.223	<0.0001	0.621
4% CO_2_	71 ± 18	45 ± 9	64 ± 23	40 ± 12	0.194	<0.0001	0.535
6% CO_2_	80 ± 21	48 ± 9	70 ± 25	43 ± 13	0.194	<0.0001	0.391
CVCi (cm/s/mmHg)							
Baseline	0.69 ± 0.24	0.46 ± 0.10	0.57 ± 0.19	0.43 ± 0.13	0.146	<0.0001	0.124
2% CO_2_	0.71 ± 0.24	0.47 ± 0.11	0.60 ± 0.24	0.43 ± 0.13	0.189	<0.0001	0.249
4% CO_2_	0.79 ± 0.24	0.48 ± 0.11	0.67 ± 0.28	0.44 ± 0.15	0.182	<0.0001	0.223
6% CO_2_	0.86 ± 0.26	0.51 ± 0.12	0.70 ± 0.26	0.45 ± 0.16	0.093	<0.0001	0.102

Cardiovascular and cerebral haemodynamic data are means ± SD. The effects of age, condition, and the age × condition interaction were assessed using a 2‐way repeated measures ANOVA. For mean arterial pressure at 6% CO_2_ young adults INDO, *n* = 34. CON, control condition before indomethacin administration; INDO, 90 min after indomethacin administration; CVCi, cerebrovascular conductance index; ETCO_2_, end‐tidal carbon dioxide; MCAv, middle cerebral artery velocity.

### 3.3 Cerebrovascular reactivity to hypercapnia

**FIGURE 1:**
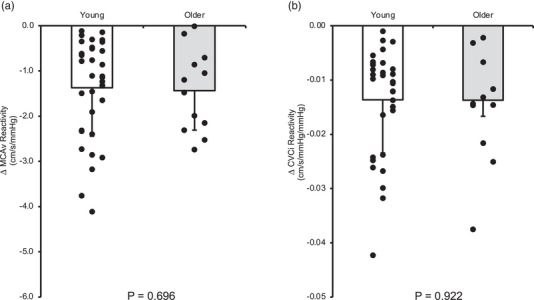
Change in cerebrovascular reactivity in young and older adults before and after indomethacin administration. (a) Change in middle cerebral artery blood velocity (ΔMCAv) reactivity and (b) change in cerebrovascular conductance index (ΔCVCi) reactivity from the control condition (CON) to 90 min after indomethacin (INDO) administration. The bar graphs show the average reactivity values for young adults in white and older adults in gray. The black dots demonstrate individual data points. Delta cerebrovascular reactivity was compared between young (*n* = 35) and older adults (*n* = 12) using a one‐way ANOVA on ranks. *P*‐values are stated on the figure.

There were no differences between age groups in MCAv reactivity either during CON (young = 2.02 ± 1.17 cm/s/mmHg vs. older = 1.98 ± 0.81 cm/s/mmHg, *P* = 0.826) or after INDO (young = 0.65 ± 0.31 cm/s/mmHg vs. older = 0.55 ± 0.44 cm/s/mmHg, *P* = 0.380). There were also no age group differences in CVCi reactivity during CON (young = 0.018 ± 0.010 cm/s/mmHg/mmHg vs. older = 0.016 ± 0.010 cm/s/mmHg/mmHg, *P* = 0.558) or after INDO (young = 0.004 ± 0.004 cm/s/mmHg/mmHg vs. older = 0.003 ± 0.004 cm/s/mmHg/mmHg, *P* = 0.166). As expected, both MCAv and CVCi reactivity were lower after INDO compared with CON in both young and older adults (*P* < 0.0001 for MCAv reactivity and *P* < 0.0001 for CVCi reactivity). However, there were no age × condition interaction effects for either MCAv reactivity (*P* = 0.862) or CVCi reactivity (*P* = 0.984). There were no differences between age groups in ΔMCAv or ΔCVCi reactivity, expressed as the difference between CON and INDO conditions (Figure 1).

### 3.4 Associations between cerebrovascular haemodynamics and prostacyclin concentration

**FIGURE 2:**
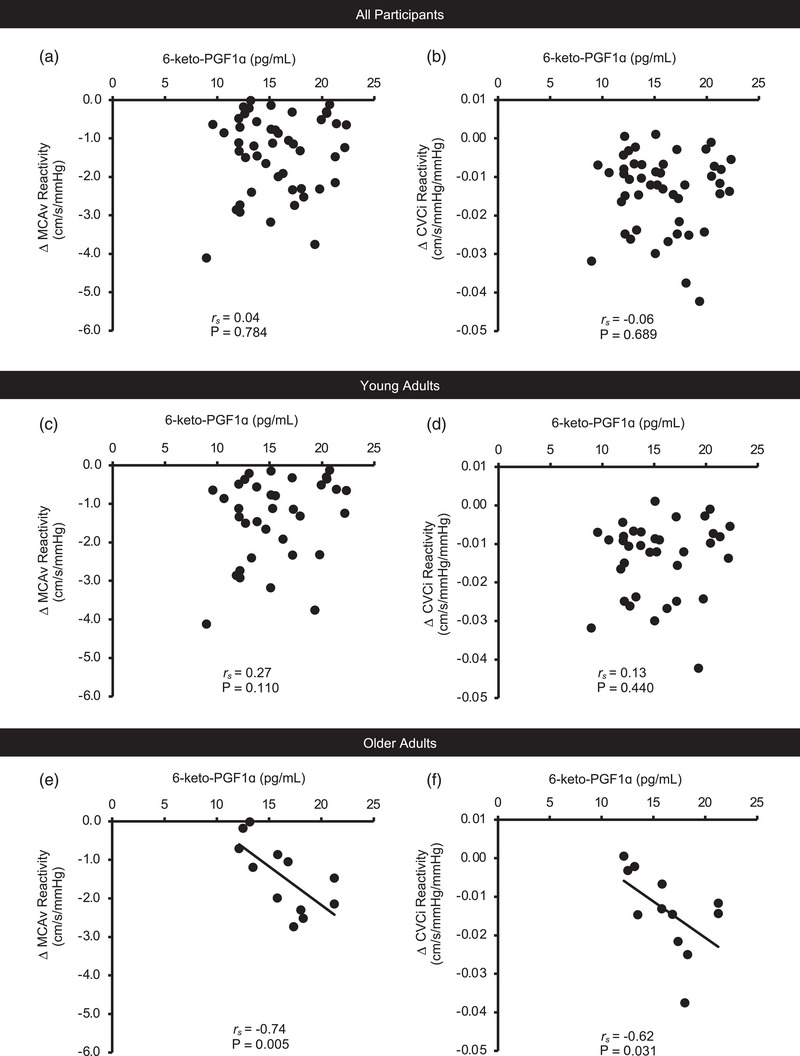
Associations between change in cerebrovascular reactivity and prostacyclin concentrations (6‐keto‐PGF1α) in all participants (a,b), young adults (c,d) and older adults (e, f). Associations between change in middle cerebral artery blood velocity (ΔMCAv) reactivity and prostacyclin (a,c,e) and associations between change in cerebrovascular conductance index (ΔCVCi) reactivity and prostacyclin (b,d,f), where change is from the control condition (CON) to 90 min after indomethacin (INDO) administration. The associations were assessed using a Spearman rank correlation in all participants (*n* = 47) young adults (*n* = 35) and older adults (*n* = 12). The continuous line indicates a significant correlation of *P* < 0.05.

There were no significant associations between prostacyclin and resting MCAv, MAP or CVCi for the CON or INDO conditions when analysed as all participants combined, young adults only, or older adults only (data not shown, *P* > 0.231 for all). There were no significant associations between prostacyclin and MCAv or CVCi reactivity during CON or after INDO when analysed as all participants combined, young adults only, or older adults only, with the exception of a positive association between prostacyclin and CVCi reactivity after INDO in young adults (Table 3). When examining the magnitude of change in reactivity after INDO, there were no significant associations between prostacyclin and ΔMCAv reactivity or ΔCVCi reactivity in all participants (Figure 2) or in the young adults (Figure 2). In contrast, in older adults, prostacyclin was inversely associated with ΔMCAv reactivity and ΔCVCi reactivity (Figure 2).

**TABLE 3:** Associations between cerebrovascular reactivity and prostacyclin concentration.

Variable	All participants (*n* = 47)		Young adults (*n* = 35)		Older adults (*n* = 12)	
CON MCAv reactivity and prostacyclin	*r*_s_ = 0.010	*P* = 0.947	*r*_s_ = −0.141	*P* = 0.415	*r*_s_ = 0.525	*P* = 0.075
INDO MCAv reactivity and prostacyclin	*r*_s_ = 0.057	*P* = 0.703	*r*_s_ = 0.268	*P* = 0.118	*r*_s_ = −0.448	*P* = 0.136
CON CVCi reactivity and prostacyclin	*r*_s_ = 0.190	*P* = 0.201	*r*_s_ = 0.093	*P* = 0.592	*r*_s_ = 0.497	*P* = 0.094
INDO CVCi reactivity and prostacyclin	*r*_s_ = 0.124	*P* = 0.403	***r*_s_ = 0.381**	***P* = 0.024**	*r*_s_ = −0.518	*P* = 0.080

The associations between cerebrovascular reactivity and prostacyclin concentration were assessed using a Spearman rank correlation in all participants, young adults, and older adults. Bolded *P*‐values are statistically significant (*P* < 0.05). CON, control condition before indomethacin administration; INDO, 90 min after indomethacin administration; CVCi, cerebrovascular conductance index; INDO, indomethacin; MCAv, middle cerebral artery blood velocity.

## 4 DISCUSSION

Our study evaluated the relationship between serum prostacyclin and cerebrovascular reactivity in healthy young and older adults, before and after cyclooxygenase inhibition. In contrast to our primary hypothesis, serum prostacyclin was not associated with cerebrovascular reactivity to hypercapnia before or after cyclooxygenase inhibition in older adults; however, older adults with higher baseline serum prostacyclin levels demonstrated a greater change in cerebrovascular reactivity between baseline and during cyclooxygenase inhibition. Additionally, we found a positive association between prostacyclin and CVCi reactivity after cyclooxygenase inhibition in young adults. Our findings suggest that older adults with higher baseline serum prostacyclin levels may rely more on cyclooxygenase products for cerebrovascular reactivity to hypercapnia. Further, the association between serum prostacyclin and CVCi reactivity after cyclooxygenase inhibition in young adults warrants further investigation.

Prostacyclin promotes vasodilatation and is an inhibitor of platelet aggregation (Arehart et al., 2007). Increased platelet aggregation has been associated with vasoconstriction and adverse cardiovascular outcomes (Angiolillo et al., 2007). Demonstrating this effect, in postmenopausal women with a history of preeclampsia, we have shown that higher platelet activation was associated with reduced cerebrovascular reactivity (Barnes et al., 2018). Additionally, previous studies have shown associations between platelet activation and other indicators of cerebrovascular and brain health. For example, in adults with a high risk for dementia, higher basal expressions of platelet activation were observed only in patients who had cognitive decline at follow‐up compared with patients without cognitive decline (Stellos et al., 2010). Furthermore, the amount of circulating thrombogenic microvesicles, which are released during platelet activation, were related to the number of white matter hyperintensities on MRI, reflecting demyelination and axonal loss in postmenopausal women. This finding suggests that elevated platelet levels are related to impairments in the brain microvasculature (Raz et al., 2013). Therefore, the impact of serum prostacyclin, and its ability to prevent platelet aggregation, may be an important consideration when evaluating the risk for declines in brain health.

In our study, we observed that older adults with higher baseline serum prostacyclin levels demonstrated a greater change in cerebrovascular reactivity before and after cyclooxygenase inhibition suggesting they may rely more on cyclooxygenase products for cerebrovascular reactivity to hypercapnia. The cerebral vasculature regulates blood flow in response to many different stimuli and through multiple different mechanisms. Although prostacyclin‐mediated vasodilatation is one of the primary mechanisms for vasodilatation in response to hypercapnia, multiple other vasodilatory stimuli, such as nitric oxide (NO), are used to promote vasodilatation during hypercapnia (Faraci et al., 1994; Lavi et al., 2003; Smith et al., 1997). The inverse association between prostacyclin and the decrease in cerebrovascular reactivity from pre‐ to post‐cyclooxygenase inhibition suggests that older adults with higher serum prostacyclin are more reliant on prostacyclin‐mediated vasodilatation. Younger adults with higher prostacyclin may have additional compensatory mechanisms to maintain cerebrovascular reactivity to hypercapnia after cyclooxygenase blockade that older adults do not have. In agreement with this idea, ageing is associated with a reduction in endothelial nitric oxide synthase expression/NO bioavailability (Brandes et al., 2005; Geary & Buchholz, 2003; Matsushita et al., 2001). Furthermore, regarding the positive association between prostacyclin and CVCi reactivity after cyclooxygenase inhibition in young adults, but not older adults, the difference in findings could be due to the differing haemodynamic effects of indomethacin on young versus older adults. For example, we have previously reported that indomethacin increases central wave reflection and augments aortic blood pressure in older adults, but not young adults (Barnes et al., 2012). One factor to note, however, is that other cyclooxygenase inhibitors do not reduce cerebral blood flow or cerebrovascular reactivity to hypercapnia to the same degree as indomethacin, despite inhibiting prostaglandin synthesis (Hoiland et al., 2016; Markus et al., 1994; Wang et al., 1993). Therefore, indomethacin may reduce cerebral blood flow and cerebrovascular reactivity through prostacyclin‐independent mechanisms, despite the inverse association between prostacyclin and the change in cerebrovascular reactivity from pre‐ to post‐indomethacin administration in older adults presented in this study. Further, there are important considerations to the studies that have reported differing cerebral blood flow responses among cyclooxygenase inhibitors, such as the participant populations, micromolar strength of the drugs and differential preferences for cyclooxygenase isomerases (Pun, 2017). Nevertheless, these findings of differing cerebral blood flow responses among cyclooxygenase inhibitors are an area of research that warrants further investigation.

Although the primary purpose of our study was not to assess age‐related differences in cerebrovascular reactivity to hypercapnia, we found no significant differences between young and older adults in cerebrovascular reactivity to hypercapnia during the control condition, after indomethacin administration, or in the magnitude of change between the two conditions. Numerous studies have investigated the influence of ageing on cerebrovascular reactivity to hypercapnia (for review, see Hoiland et al., 2019). There is considerable interest in the variability of age‐related changes in cerebrovascular reactivity, particularly in older adults. This variability could be due to genetics, habitual physical activity levels (Miller et al., 2018), or a cumulation of lifestyle factors, including the use of daily aspirin. Based on the Godin–Shepherd Leisure‐Time Physical Activity Questionnaire results, the adults in the present study were also considered habitually active and were without underlying cardiovascular disease. The present study provides an additional factor, prostacyclin, that may explain some of the variability in age‐related changes in cerebrovascular reactivity. Adding to potential sources of variation, the present study included participants of both sexes but did not assess the association between serum prostacyclin and cerebrovascular reactivity by sex, as we were underpowered to do so. This would be an important assessment in future studies, as it is possible that the cyclooxygenase contribution to cerebral blood flow responses varies by sex and/or menstrual cycle phase (Peltonen et al., 2016).

There are several limitations of the present study. Though a longitudinal approach is preferable to study ageing, our study utilized a cross‐sectional study design to improve feasibility. We were interested in the association between serum prostacyclin and cerebrovascular reactivity before and after cyclooxygenase inhibition via indomethacin. As mentioned, other cyclooxygenase inhibitors do not reduce cerebral blood flow to the same degree as indomethacin, and it is possible that indomethacin reduces cerebral blood flow through prostacyclin‐independent mechanisms. However, indomethacin is commonly used to measure cerebral blood flow or cerebral artery blood velocity responses in response to cyclooxygenase inhibition (Bain et al., 2016; Ivancev et al., 2009; Kellawan et al., 2020; Peltonen et al., 2016). In addition, other cyclooxygenase products such as thromboxane may be important to consider in future studies. Another limitation is that this study was not a randomized placebo design. However, our previous work has examined the influence of indomethacin on cerebrovascular reactivity to CO_2_, and the purpose of the present study was not to repeat these studies using a placebo arm, but to evaluate the influence of circulating prostacyclin. Indomethacin was utilized as a tool to inhibit prostacyclin production to help further explore this relationship. Using TCD ultrasound to measure cerebral blood flow is a limitation, as it relies on the assumption that the middle cerebral artery does not vasodilate during hypercapnia. Despite this assumption, TCD is still commonly used to evaluate cerebral artery blood velocity responses to stimuli as it provides the temporal resolution to evaluate beat‐to‐beat changes in cerebrovascular haemodynamics. Further, the influence of cyclooxygenase inhibition, or the interaction between cyclooxygenase inhibition and CO_2_, on middle cerebral artery diameter is unclear. Finally, the blood draws to assess prostacyclin levels were collected on a separate day from the experimental protocol, as some of the blood collected was used for analyses to determine participant eligibility. Importantly, both of the laboratory visits occurred under similar conditions (e.g., fasted in the morning and after having abstained from exercise); therefore, potential sources of variability were likely minimized.

In conclusion, we report an association between serum prostacyclin and the magnitude of change in cerebrovascular reactivity to hypercapnia before and after cyclooxygenase inhibition in older adults. Our results suggest that older adults with higher serum prostacyclin may be more reliant on prostacyclin‐mediated vasodilatation and/or may lack the compensatory mechanisms for cerebral vasodilatation present in younger adults with elevated serum prostacyclin. Future studies should evaluate how platelet activation or reductions in circulating prostacyclin may contribute to age‐related changes in cerebrovascular function, especially in those with elevated platelets or low prostacyclin concentrations.

## AUTHOR CONTRIBUTIONS

Experiments were performed in the Bruno Balke Biodynamics Laboratory under the direction of Dr. Jill Barnes at the University of Wisconsin‐Madison. Conception or design of the work: Adam T. Corkery, Kathleen B. Miller, Jill N. Barnes; acquisition, analysis, or interpretation of data for the work: Adam T. Corkery, Kathleen B. Miller, Carissa A. Loeper, Laura H. Tetri, Andrew G. Pearson, Nicole A. Loggie, Anna J. Howery, Marlowe W. Eldridge, Jill N. Barnes; drafting of the work or revising it critically for important intellectual content: Adam T. Corkery, Kathleen B. Miller, Carissa A. Loeper, Laura H. Tetri, Andrew G. Pearson, Nicole A. Loggie, Anna J. Howery, Marlowe W. Eldridge, Jill N. Barnes. All authors (Adam T. Corkery, Kathleen B. Miller, Carissa A. Loeper, Laura H. Tetri, Andrew G. Pearson, Nicole A. Loggie, Anna J. Howery, Marlowe W. Eldridge, and Jill N. Barnes) approved the final version of the manuscript and agree to be accountable for all aspects of the work in ensuring that questions related to the accuracy or integrity of any part of the work are appropriately investigated and resolved. All persons designated as authors qualify for authorship, and all those who qualify for authorship are listed.

## CONFLICT OF INTEREST

The authors have no conflicts of interest to disclose.

## Data Availability

The data that support the findings of this study are available on request from the corresponding author. The data are not publicly available due to privacy or ethical restrictions.
